# Supplementary material for: Molecular determinants of Yellow Fever Virus pathogenicity in Syrian Golden Hamsters: one mutation away from virulence
Source: Emerg Microbes Infect. 2018 Mar 29;7:51. doi: 10.1038/s41426-018-0053-x (PMC5874243; doi:10.1038/s41426-018-0053-x)
Supplement: Supplementary file 1 — Supplementary materials(PDF 207 kb) [file 41426_2018_53_MOESM1_ESM.pdf]

SUPPLEMENTARY MATERIALS

Supplementary Protocol S1

Cells. Baby hamster kidney BHK21 (BHK21) cells (ATCC, number CCL10) were grown at 37°C with 5% CO2 in Minimum Essential Medium (Earle's Salts, LifeTechnologie) with 2.5% fetal bovine serum (FBS; Life Technologies), 1% L-Glutamine (200 mM; Life Technologies), 2,5% tryptose phosphate broth (TPB; Life Technologie) and 1% Penicillin/Streptomycin (5000U/mL and 5000 µg/mL; LifeTechnologie).

Tissue-culture infectious dose 50 (TCID50) assay. For each determination, a 96-well plate culture containing 10^4 BHK-21 cells in 100µL of culture medium per well was inoculated with 100µL of serial 10-fold dilutions of clarified viral culture supernatants (in culture medium without FBS). Each viral supernatant was tested on a half-plate with 6 successive dilutions in quadruplicate with 6 uninfected wells. The plates were incubated for 7 days and for each well, 100µL of supernatant was extracted using the QiaCube HT device (see corresponding section) and tested using a YFV-specific qRT-PCR method (see corresponding section). The default positivity limit was set to a Ct of 30. TCID50 determination was performed using the method of Reed & Muench (1).

Preparation of cDNA fragments. Subgenomic DNA fragments were synthesized de novo (Genscript) and amplified by High Fidelity PCR using the Platinum PCR SuperMix High Fidelity kit (Life Technologies) and 8 sets of primers (see Table in supplementary Table S1). The mixture (final volume: 50µL) consisted in 45µL of SuperMix, 2µL of DNA template (5ng/µL) and 200nM of each primer. PCR was performed on a Thermocycler (2720 Thermal Cycler, Applied Biosystems(AB)) with the following conditions: 2min at 94°C followed by 40 cycles of 15sec at 94°C, 30sec at 55°C, 4min at 68°C and a final step of 5min at 68°C.

Transfection and stock production. A final amount of 1µg of an equimolar mix of the subgenomic cDNA fragments amplified by PCR was incubated with 10.5µL of Lipofectamine 3000 and 2µL of transfection reagent in 0.25mL of Opti-MEM medium (all from Life Technologies). According to the manufacturer's instructions, the mixture was added to a 25 cm^2 culture flask of subconfluent cells containing 1mL of culture medium without antibiotics. After an incubation of 24h, the cell supernatant was removed, cells were washed twice in Hank's balanced salt solution (HBSS; Life Technologies) and 5mL of fresh culture medium was added. The cell supernatants were harvested at 9 days post-transfection, buffered with 4-(2-hydroxyethyl)-1-piperazineethanesulfonic acid (HEPES, Life Technologies) at a final concentration of 2.5%, clarified by centrifugation (2800g, 5 min), aliquoted and stored at -80 °C. Each virus was then passaged two times onto BHK21 cells. First and second passages were performed by inoculating 100µL (and 10µL respectively) of clarified cell supernatant onto cells in a 25 cm^2 culture flask containing 1mL of culture medium without FBS. After adsorption of the virus for 1h, cells were washed twice in HBSS and 5mL of fresh culture medium with 2,5% FBS was added. After 72 hours (2nd passage) to 5 days (1st passage) of incubation at 37°C, cell supernatants were harvested, buffered with HEPES (2.5%), clarified by centrifugation (2800g, 5 min), aliquoted and stored at -80 °C. Clarified cell supernatants from the second passage (virus stocks) were used to perform viral RNA quantification, TCID50 assays and whole-genome sequencing (see corresponding sections).

Quantitative real-time RT-PCR assays. Quantitative real-time PCR (qRT-PCR) assays were performed using the EXPRESS SuperScript kit for One-Step qRT-PCR (Invitrogen). The reaction mix (final volume: 10µL) was prepared as follows: 5 µL of EXPRESS SuperScript® qPCR SuperMix Universal with ROX, 1 µL of EXPRESS SuperScript® Mix for One-Step qPCR, sense and reverse primers (final concentration: 0.5µM), probe (final concentration: 0.2µM) and 2.5µL of extracted nucleic acids. qRT-PCR were performed on QuantStudio 12K Flex Real-Time PCR system (ThermoFisher) with different cycling conditions for YFV detection (5min at

45°C, 2min at 94°C, then 40 cycles of 01sec at 94°C and 20sec at 60°C with data collection) and Actin detection (5min at 50°C, 2min at 94°C, then 6 cycles of 03sec at 94°C and 30sec at 63°C with data collection, and finally 40 cycles of 03sec at 94°C and 30sec at 60°C with data collection). Primers and probe sequences are detailed in Table in supplementary Table S2.

Samples collection. Livers were sampled from euthanized hamsters and ground in 50mL polypropylene tubes (Labcon, PerformR tubes) with 5 mL of HBSS, 3 mm Tungsten Carbide beads (Qiagen) using a grinder (Mixer Mill MM 400, Retsch) for 4min 30sec with a frequency of 27s^{-1}. Liver homogenates were then centrifugated for 5min at 2100g, aliquoted and stored at -80°C. Liver homogenates (200µL) were treated with 50µL of proteinase K (PK) (Macherey-Nagel) for 1h 40min at 57°C before nucleic acid extraction using either the EZ1 Biorobot or the QiaCube HT device (see corresponding section).

High fidelity RT-PCR amplification. High fidelity RT-PCR amplification was performed using the SuperScript III Platinium One-Step RT-PCR kit (SSIII, Invitrogen) following the manufacturer's instructions. The reaction mix (final volume: 25µL) was prepared as follow: 25µL of 2X MasterMix, 2µL of SuperScript™ III RT/Platinum™ Taq Mix, 1µL of both primers (10µM), 17µL of distilled water and 3µL of extracted nucleic acids. RT-PCR was performed on a Thermocycler (2720 Thermal Cycler, Applied Biosystems(AB)) with the following conditions: 30min at 50°C, 2min at 94°C followed by 40 cycles of 15sec at 94°C, 30sec at 55°C, 4min at 68°C and a final step of 5min at 68°C. The sets of primers used for this amplification step are detailed in the Table in supplementary Table S3.

Sequence analysis. Both whole and partial coding nucleotide sequences were determined using next-generation sequencing methods: 5 (or 2) overlapping amplicons spanning either the complete genome sequence or the first part of the genome were produced from the extracted

RNA (see corresponding section) using the SuperScript® III One-Step RT-PCR System with Platinum®Taq High Fidelity kit (Invitrogen) and specific primers. PCR products were purified on 96-wells purification plates (NucleoFast® 96 PCR Plate, Macherey-Nagel) following the manufacturer's instructions. DNA concentration was measured using a Nanodrop (Nanodrop 1000 Spectrophotometer, ThermoScientific) and fragments were pooled in equimolar proportions for library building. Sequencing was performed using the PGM Ion torrent technology (Thermo Fisher Scientific) following the manufacturer's instructions. Automated read datasets provided by Torrent software suite 5.0.2 were trimmed according to quality score (99%) and length (reads shorter than 30 bp were removed) using CLC genomics workbench software (CLC bio-Qiagen). Primers used for RT-PCR were removed using an in-house software package. Remaining reads were mapped using the expected sequence of the virus as a reference using CLC and a de novo contig was produced to ensure that the consensus sequence was not affected by the reference sequence.

References

1. UniProt: the universal protein knowledgebase. *Nucleic Acids Res.* 45: D158-D169 (2017)
2. Aubry F, Nougairede A, de Fabritus L, Querat G, Gould EA, de Lamballerie X. Single-stranded positive-sense RNA viruses generated in days using infectious subgenomic amplicons. J Gen Virol. 2014;95(Pt 11):2462-7.
3. ReedLJ, Muench (1938) A simple method of estimating fifty per cent endpoints. AmJ Hyg 27: 493–497
