# Supplementary material for: Molecular determinants of Yellow Fever Virus pathogenicity in Syrian Golden Hamsters: one mutation away from virulence
Source: Emerg Microbes Infect. 2018 Mar 29;7:51. doi: 10.1038/s41426-018-0053-x (PMC5874243; doi:10.1038/s41426-018-0053-x)
Supplement: Supplementary file 2 — Supplementary Table S1(PDF 30 kb) [file 41426_2018_53_MOESM2_ESM.pdf]

Fragment	Primer name	Sequence	Genome position (nt)	Size (nt)
**FI**	Sens 2517	cacccaactgatcttcagcatct	49-72 (pCMV)	4038
	Rev ext I	ctatgataaccacggtacaaaagag	3895-3919	
**FI.1**	Sens 2517	cacccaactgatcttcagcatct	49-72 (pCMV)	2145
	FI.1 Rev	tgtgccactggtaagtgaga	2007-2026	
**FI.2**	FI.2 Sens	accaatgatgatgaagtgctg	1927-1947	1992
	Rev ext I	ctatgataaccacggtacaaaagag	3895-3919	
**FI.1.1***	FI.1.1 Sens	ggccggccgccagcgg	0-16 (pCMV)	1053 (+758 pCMV)
	FI.1.1 Reverse	tgctccagggtagctgaaac	916-935	
**FI.1.2***	FI.1.2 Sens Asibi	gtttcagctaccctggagcaagac	916-939	426
	FI.1.2 Sens E27	gtttcagctaccctggagcacgac		
	FI.1.2 Sens E27-E28	gtttcagctaccctggagcacggc		
	FI.1.2 Reverse Asibi	catcaaacttgagagtcttaatgtcgg	1316-1342	
	FI.1.2 Reverse E155	catcaaacttgagagtcttaatggcgg		
**FI.1 bis****	FI.1.1 Sens fusion	gaataagggcgacacggaaatgtcac	26-52 (pCMV)	1342 (+758 pCMV)
	FI.1.2 Reverse fusion	catcaaacttgagagtcttaatg	1320-1342	
**F1.2bis**	FI.2 bis Sens Asibi	aaaattggaataccgacattaag	1304-1326	2615
	FI.2 bis Sens E155	aaaattggaataccgccattaag		
	Rev ext I	ctatgataaccacggtacaaaagag	3895-3919	
**FII**	Sens int II	catggctctgttgacacctgtc	3843-3864	3017
	Rev ext II	ccggccaactccagggtgaagc	6838-6859	
**FIII**	Sens int III	gctggagaaaaccaaagaggacc	6783-6805	4203
	Rev ext III (R292)	tactggaacgttgtgagggtaaac	synthetic sequence following pA signal	

Table S1. Subgenomic fragments and correspondding primers used for virus production using the ISA method (2).
