# Supplementary material for: Molecular determinants of Yellow Fever Virus pathogenicity in Syrian Golden Hamsters: one mutation away from virulence
Source: Emerg Microbes Infect. 2018 Mar 29;7:51. doi: 10.1038/s41426-018-0053-x (PMC5874243; doi:10.1038/s41426-018-0053-x)
Supplement: Supplementary file 3 — Supplementary Table S2(PDF 93 kb) [file 41426_2018_53_MOESM3_ESM.pdf]

Name	Size (nt)	Nucleotide sequence	Hybridation site
Yellow Fever Forward	25	AATCGAGTTGCTAGGCAATAAACAC	40-64
Yellow Fever Reverse 2	21	TCCCTGAGCTTTACGACCAGA	122-142
Yellow Fever Probe	23	ATCGTTCGTTGAGCGATTAGCAG	81-103
Actin Forward	21	GTSTGGATYGGHGGHTCBATC	1015-1035
Actin Reverse	21	GAYTCRTCRTAYTCCTSCTTG	1074-1094
Actine Probe	21	ACCTTCCAGCAGATGTGGATC	1051-1071

Table S2. Yellow Fever Virus and Actin-specific qRT-PCR systems.
