# Supplementary material for: Molecular determinants of Yellow Fever Virus pathogenicity in Syrian Golden Hamsters: one mutation away from virulence
Source: Emerg Microbes Infect. 2018 Mar 29;7:51. doi: 10.1038/s41426-018-0053-x (PMC5874243; doi:10.1038/s41426-018-0053-x)
Supplement: Supplementary file 4 — Supplementary Table S3(PDF 28 kb) [file 41426_2018_53_MOESM4_ESM.pdf]

Name	Size (nt)	Nucleotide sequence	Hybridation site
Asibi_IT_S0	20	ATGTCTGGTCGTAAAGCTCAG	1-21
Asibi_IT_R0.1	21	ACTGTCTCTAGTGAGATGTCC	978-998
Asibi_IT_S0.2	20	TGGAGGAACTTGGGTTTCAG	903-922
Asibi_IT_R0	20	CTTCCCTCTTTGTGCCACTG	2017-2036
Asibi_IT_S1	21	CCTCAACCAATGATGATGAAG	1922-1942
Asibi_IT_R1.1	21	CTTCAACTGATGTTCCAATCG	3026-3046
Asibi_IT_S1.2	20	TTACAAGGAGTGTGAGTGGC	2994-3013
Asibi_IT_R1	20	CACCTCAGCCATAGTGACAG	3860-3879
Asibi_IT_S2	22	ATCTAAATGCAGTTTCTCTCTG	3764-3785
Asibi_IT_R2	20	AGTCATGCCCTGTGTTCCAG	5496-5515
Asibi_IT_S3	21	CCTGGGACTAGTGATGAATTT	5422-5442
Asibi_IT_R3	20	GTTCCCTCTTCCAGACTTCA	7545-7564
Asibi_IT_S4	21	GTGGGAGTCATGTACAATCTA	7474-7494
Asibi_IT_R4	20	TCAGATAAGCTCACCCGGTT	10217-10236

Table S3. Yellow Fever Virus-specific PCR systems used for High-Fidelity RT-PCR amplification.
