# Supplementary material for: Molecular determinants of Yellow Fever Virus pathogenicity in Syrian Golden Hamsters: one mutation away from virulence
Source: Emerg Microbes Infect. 2018 Mar 29;7:51. doi: 10.1038/s41426-018-0053-x (PMC5874243; doi:10.1038/s41426-018-0053-x)
Supplement: Supplementary file 5 — Supplementary Table S4(PDF 111 kb) [file 41426_2018_53_MOESM5_ESM.pdf]

Protein	AA position within YFV (AY640589) polyprotein sequence
Capside	1-101
ER anchor	102-121
pr	122-210
M	211-285
Env	286-778
NS1	779-1130
NS2A	1131-1354
NS2A a	1131-1320
NS2B	1355-1484
NS3	1485-2107
NS4A	2108-2233
2k	2234-2256
NS4B	2257-2506
NS5	2507-3411

Table S4. Mature proteins positions within Yellow Fever Virus polyprotein sequence (obtained from UniProt database, www.uniprot.org,(1)).
