# Supplementary material for: Molecular determinants of Yellow Fever Virus pathogenicity in Syrian Golden Hamsters: one mutation away from virulence
Source: Emerg Microbes Infect. 2018 Mar 29;7:51. doi: 10.1038/s41426-018-0053-x (PMC5874243; doi:10.1038/s41426-018-0053-x)
Supplement: Supplementary file 6 — Supplementary Table S5(PDF 104 kb) [file 41426_2018_53_MOESM6_ESM.pdf]

	Nucleotides		Amino Acids		
**Nucleotide/Codon position in CDS**	Asibi sequence	Ap7M sequence	Position in protein*	Asibi sequence	Ap7M sequence
684	A	G	prM107	K	*k*
769	C	U	prM136	L	*l*
882	G	A	E9	R	*r*
936	A	C	E27	Q	H
938	A	G	E28	D	G
1319	A	C	E155	D	A
1823	A	G	E323	K	R
1847	A	G	E331	K	R
2661	U	C	NS1109	D	*d*
3703	A	G	NS2A48	T	A
682	CAC	GCA	E27	Q	A
682	CAC	AAC	E27	Q	N
1314	ACC	GCC	E154	T	A

Table S5. Description of Ap7M, reversion, positive and functional mutant strains nucleotide sequences.

*The Amino Acid (AA) position is given with reference to the beginning of mature protein sequence (ie. AA position 107 in prM, written prM107, corresponds to AA position 228 within the precursor polyprotein). A detailed map of protein positions within the YFV polyprotein sequence is provided as supplementary Table S4. Synonymous mutations are indicated by grey, italic, lowercase letters. For each strain, the AA type encountered at a given position is indicated by the corresponding AA single letter code.
